# Supplementary material for: Novel Driver Strength Index highlights important cancer genes in TCGA PanCanAtlas patients
Source: PeerJ. 2022 Aug 11;10:e13860. doi: 10.7717/peerj.13860 (PMC9375969; doi:10.7717/peerj.13860)
Supplement: Supplemental Information 2 [file peerj-10-13860-s002.zip › COHORTS/genes plots/cohorts/2021_8_16_14_9_distribution_cohorts_males_ACC.pdf]

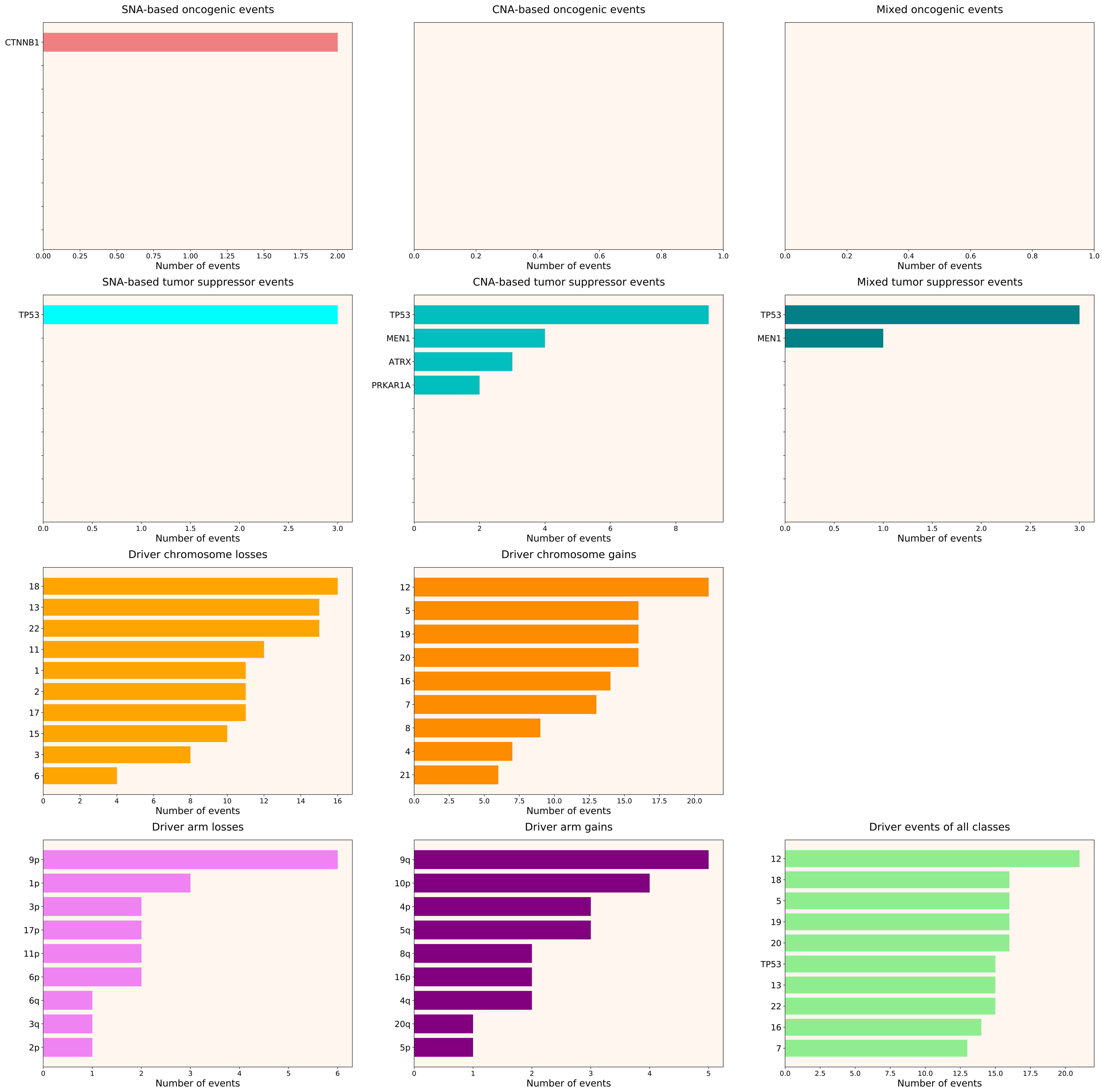
SNA-based oncogenic events
CTNNB1
Number of events
CNA-based oncogenic events
Number of events
Mixed oncogenic events
Number of events
SNA-based tumor suppressor events
TP53
Number of events
CNA-based tumor suppressor events
TP53
MEN1
ATRX
PRKAR1A
Number of events
Mixed tumor suppressor events
TP53
MEN1
Number of events
Driver chromosome losses
18
13
22
11
1
2
17
15
3
6
Number of events
Driver chromosome gains
12
5
19
20
16
7
8
4
21
Number of events
Driver arm losses
9p
1p
3p
17p
11p
6p
6q
3q
2p
Number of events
Driver arm gains
9q
10p
4p
5q
8q
16p
4q
20q
5p
Number of events
Driver events of all classes
12
18
5
19
20
TP53
13
22
16
7
Number of events
